# Special Issue: “Molecular Research in Human Microbiome 2.0”

**DOI:** 10.3390/ijms262311379

**Published:** 2025-11-25

**Authors:** Maria Teresa Mascellino

**Affiliations:** Department of Public Health and Infectious Diseases, Sapienza University, Piazzale Aldo Moro 5, 00185 Rome, Italy; mariateresa.mascellino@uniroma1.it; Tel.: +39-3281685935; Fax: +39-0649972526

The human microbiome is crucial to the health and welfare of the global population; a healthy microbiome protects us against pathogens while preserving beneficial organisms, assists immune system development and generally maintains a good level of well-being. Disorders of the human microbiome are associated with diseases ranging from the non-neoplastic to the tumorigenic, including cancer, inflammation and intestinal damage [1], as well as other pathologies such as obesity, depression and anxiety [2,3]. Furthermore, many studies have identified a relationship between microbiome composition and the brain, as well as the essential role of the intestine–brain axis in the pathogenesis and evolution of many diseases such as HD (Huntington’s disease) [4,5].

Based on innovative applications of omics technology, the mechanisms underlying the role of the microbiome in human pathogenesis and diseases have been explored, highlighting changes in the occurrence of many diseases [6]. Next-generation sequencing platforms, the integration of multi-omics data, and bioinformatics development have improved our knowledge of gut microbial composition [7].

Another technique, Loop-Mediated Isothermal Amplification (LAMP), also offers alternative options for combating drug resistance via the rapid and sensitive detection of carbapenemase genes in CRE (carbapenem-resistant *Enterobacterales*) [8].

Metabolomic technologies may provide critical information about the role of the gut microbiome in many diseases, especially in cancer [9]. In the first contribution to this Special Issue, Jaye K et al. (contribution 1)underscore the anti-proliferative effects of three key gut microbial metabolites—sodium butyrate, inosine, and nisin—against the MCF7 and MDA-MB-231 breast adenocarcinoma cell lines and the MCF10A normal breast epithelial cell line, demonstrating their anti-proliferative action against breast adenocarcinoma cells through proteomics analyses. The role of the gut microbiota in the treatment of breast cancer is a relatively new area of oncological research, and may be a good alternative to drugs which are cytotoxic to normal breast tissue due to their lack of selectivity—a serious concern in anticancer drug discovery. The results underline the effectiveness of these compounds against breast cancer, though their possible toxicity should always be taken into consideration. However, knowledge in this field is still far from certain. The increased production of ROS within a host can lead to carcinogenesis due to DNA damage in normal cells and consequent apoptosis, the latter of which is a desirable feature for prospective anticancer agents.

The second contribution to this Special Issue concerns the presence of the red microbial complex (*P. gingivalis*, *T. denticola* and *T. forsythia*) in the oral microflora of older Japanese individuals, and the relationships between these and other oral bacteria (contribution 2). In this case, interference between the red complex and the normal bacterial population was observed, which affected the oral microflora’s composition in these subjects and led to pathologies and changes in the mouth microenvironment. The red complex is a major group of bacteria associated with the progression of periodontal diseases that may cause microbial shifts, demonstrating the importance of this study.

The gut microbiota–brain axis, and its relationship with neurodegenerative diseases, are frequently reported in the literature [10,11]. Dysbiosis in the gastrointestinal (GI) tract is frequently observed in individuals with neurological disorders, contributing to the worsening of their symptoms [12]. In the third contribution to this Special Issue, Moreno and Ashwood (contribution 3) analyze how autism is correlated with gut dysbiosis. In recent years, greater attention has been paid to the impact of microbial manipulation on outcomes of autism. The authors reported different approaches to microbial interference, such as antibiotic therapy, fecal transplantation, prebiotics and probiotics, which were particularly effective at reducing the severity of both autism and gastrointestinal symptoms. Overall, this article stresses the crucial importance of the gut microbiota in host health, and its influence on the central nervous system, reaffirming the influence of the gut microbiota–brain axis in psychiatric disorders.

To expand on the previous article, Bauch and Baur’s study (contribution 4), concerning decreases in cognitive capacity in patients with gut dysbiosis, further confirms the strong link between the gut and brain [4,13]. The authors introduced interference to functional gut microbiome data from 100 healthy controls to predict the progression from normal cognition to MCI (mild cognitive impairment) over a 4-year follow-up period, allowing for early detection of subjects at risk of precocious Alzheimer’s disease (AD). Altered gut microbiome composition is well-documented in cross-sectional studies of patients with AD, and even in patients with preclinical AD; this article therefore underlines the predictive value of gut microbiome models in this earlier stage of disease, crucial for the correct and early diagnosis of Alzheimer’s pathology.

In the final contribution to this Special Issue, the presence and function of a possible microbiome present in the dental pulp is considered (contribution 5). For several decades, the pulp tissues of healthy teeth have been considered a sterile environment; however, the existence of an apparent core pulpal microbiome has been reported. The authors of this study identified DNA (mainly bacterial DNA) in all pulp samples, but also in all dentin samples and negative controls, likely indicating reagent contamination. This was supported by the fact that the majority of bacteria detected in these samples belonged to the genera *Burkholderia*, which is a common contaminant species. The fact that the presence of bacterial DNA was found in all specimens of healthy pulp does not seem to support the definite presence of a core microbiome in these samples. Further detailed molecular studies are necessary in this field.

In conclusion, much still needs to be investigated in this field. Dysbiosis is linked to a wide range of health issues, including metabolic abnormalities, inflammatory bowel diseases and mental disorders (Figure 1). This underscores the vital role of the gut microbiota in maintaining overall physiological stability [14,15]. The potential anticancer properties of different groups of gut metabolites play a fundamental role and should be further investigated in various types of cancer [16]; gut microbiome alteration is a crucial factor in the development of tumors, particularly colorectal cancer [17]. Moreover, the use of antibiotics, probiotics, prebiotics and dietary changes can be used to restore microbial balance, underlining the significance of microbial communities in controlling immune responses and metabolic processes.

## Figures and Tables

**Figure 1:**
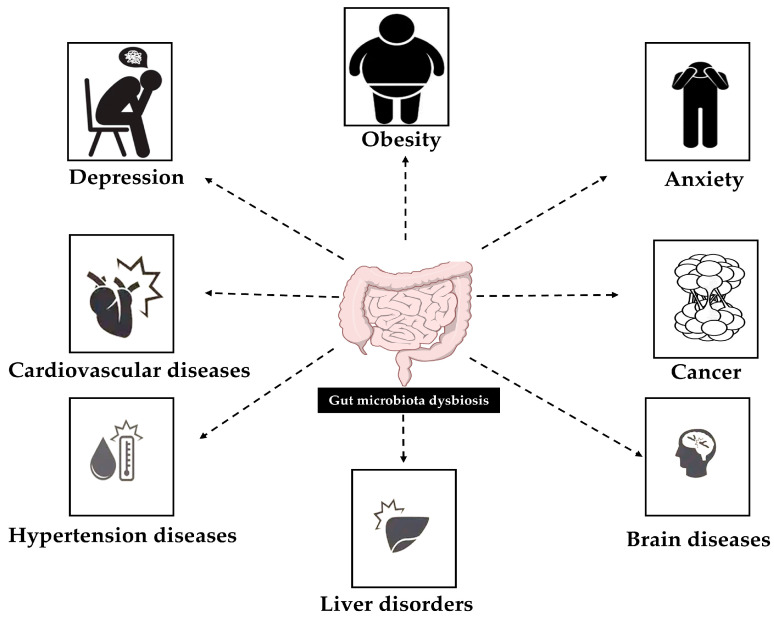
The impact of gut microbiota dysbiosis on human health, showing influence on the brain and mental health in addition to cancer development and other pathologies linked to the oral microflora.

## References

[1] Mascellino M.T. (2023). Molecular Research in Human Microbiome.

[2] Simpson C.A., Diaz-Arteche C., Eliby D., Schwartz O.S., Simmons J.G., Cowan C.S.M. (2020). The Gut Microbiota in Anxiety and Depression—A Systematic Review. Clin. Psychol. Rev..

[3] Xu Z., Jiang W., Huang W., Lin Y., Chan F.K.L., Ng S.C. (2022). Gut Microbiota in Patients with Obesity and Metabolic Disorders—A Systematic Review. Genes Nutr..

[4] Margolis K.G., Cryan J.F., Mayer E.A. (2021). The Microbiota-Gut-Brain Axis: From Motility to Mood. Gastroenterology.

[5] Wronka D., Karlik A., Misiorek J.O., Przybyl L. (2023). What the Gut Tells the Brain—Is There a Link between Microbiota and Huntington’s Disease?. Int. J. Mol. Sci..

[6] Candel S., Tyrkalska S.D., Pérez-Sanz F., Moreno-Docón A., Esteban Á., Cayuela M.L., Mulero V. (2023). Analysis of 16S rRNA Gene Sequence of Nasopharyngeal Exudate Reveals Changes in Key Microbial Communities Associated with Aging. Int. J. Mol. Sci..

[7] Papadaki E., Kakkos I., Vlamos P., Petropoulou O., Miloulis S., Vrahatis A. (2025). Recent Web Platforms for Multi-Omics Integration Unlocking Biological Complexity. Appl. Sci..

[8] Poirier A.C., Kuang D., Siedler B.S., Borah K., Mehat J.W., Liu J., Tai C., Wang X., Poirier A.C., van Vliet A.H.M. (2022). Development of Loop-Mediated Isothermal Amplification Rapid Diagnostic Assays for the Detection of *Klebsiella pneumoniae* and Carbapenemase Genes in Clinical Samples. Front. Mol. Biosci..

[9] Saud Hussein A., Ibraheem Salih N., Hashim Saadoon I. (2021). Effect of Microbiota in the Development of Breast Cancer. Arch. Razi Inst..

[10] Mitrea L., Neme¸s S.A., Szabo K., Teleky B.E., Vodnar D.C. (2022). Guts imbalance imbalances the brain: A review of gut microbiota association with neurological and psychiatric disorders. Front. Med..

[11] Góralczyk-Bińkowska A., Szmajda-Krygier D., Kozłowska E. (2022). The Microbiota-Gut-Brain Axis in Psychiatric disorders. Int. J. Mol. Sci..

[12] Mazzone L., Dooling S.W., Volpe E., Uljarevi’c M., Waters J.L., Sabatini A., Arturi L., Abate R., Riccioni A., Siracusano M. (2023). Precision microbial intervention improves social behavior but not autism severity: A pilot double-blind randomized placebo-controlled trial. Cell Host Microbe.

[13] Gates E.J., Bernath A.K., Klegeris A. (2022). Modifying the diet and gut microbiota to prevent and manage neurodegenerative diseases. Rev. Neurosci..

[14] Oliva A., Aversano L., De Angelis M., Mascellino M.T., Miele M.C., Morelli S., Battaglia R., Iera J., Bruno G., Corazziari E.S. (2019). Persistent Systemic Microbial Translocation, Inflammation, and Intestinal Damage During *Clostridioides difficile* Infection. Open Forum Infect. Dis..

[15] Zhang Q., Liu Y., Li Y., Bai G., Pang J., Wu M., Li J., Zhao X., Xia Y. (2025). Implications of Gut Microbiota-Mediated Epigenetic Modifications in Intestinal Diseases. Gut Microbes.

[16] Mascellino M.T. (2023). Molecular Research in Human Microbiome. Int. J. Mol. Sci..

[17] Dewan A., Tattoli I., Mascellino M.T. (2025). The Impact of *Fusobacterium nucleatum* and the Genotypic Biomarker KRAS on Colorectal Cancer Pathogenesis. Int. J. Mol. Sci..

